# The Evaluation of Root Fracture with Cone Beam Computed Tomography (CBCT): An Epidemiological Study

**DOI:** 10.4317/jced.54009

**Published:** 2018-01-01

**Authors:** Mehmet-Sinan Doğan, Michele Callea, Lindawati S. Kusdhany, Ahmet Aras, Diah-Ayu Maharani, Masita Mandasari, Melissa Adiatman, Izzet Yavuz

**Affiliations:** 1 Harran University, Faculty of Dentistry, Department of Pediatric Dentistry,Şanlıurfa, Türkiye; 2 Unit of Dentistry, Bambino Gesù Children’s Hospital, IRCCS, Rome, Italy; 3 Prosthodontic Department, Faculty of Dentistry, Universitas Indonesia; 4 Oral and Tooth Health Center, Department of Pediatric Dentistry, Diyarbakir, Türkiye; 5 Department of Preventive and Public Health Dentistry, Faculty of Denstistry, Universitas Indonesia; 6 Departement of Oral Medicine, Faculty of Dentistry, Universitas Indonesia, Jakarta, Indonesia

## Abstract

**Background:**

The aim of this study was evaluation of the cone-beam computed tomography (CBCT) image of 50 patients at the ages of 8-15 suspecting root fracture and root fracture occurred, exposed to dental traumatic. In additionally, this study was showed effect of crown fracture on root fracture healing.

**Material and Methods:**

All of the individuals included in the study were obtained images with the cone-beam computed tomography range of 0,3 voxel and 8.9 seconds.(i-CAT®, Model 17-19, Imaging SciencesInternational, Hatfield, Pa USA).The information obtained from the history and CBCT images of patients were evaluated using chi-square test statistical method the mean and the distribution of the independent variables.

**Results:**

50 children, have been exposed to trauma, was detected root fracture injury in 97 teeth. Horizontal root fracture 63.9% of the 97 tooth, the oblique in 31.9%, both the horizontal and oblique in 1.03%, partial fracture in 2.06% ,and both horizontally and vertical in 1.03% was observed.The most affected teeth, respectively of, are the maxillary central incisor (41.23% left, right, 37.11%), maxillary left lateral incisor (9.27%), maxillary right lateral incisor (11.34%), and mandibular central incisor (1.03%).

**Conclusions:**

Crown fractures have negative effects on spontaneous healing of root fractures. CBCT are used selected as an alternative to with conventional radiography for diagnosis of root fractures. In particular, ıt’s cross-sectional image is quite useful and has been provided more conveniences seeing the results of diagnosis and treatment for clinician.

** Key words:**Root fracture, CBCT, Epidemiolog.

## Introduction

Popularization of preventive dentistry applications, the frequency of dental caries and periodontal problems in children decreased, but epidemiological researches showed that trauma-based dental injuries are still serious problems in children (1-3). Root fractures are less widespread compared to other injuries in classification dental-trauma. Root fractures represent 0.5-7% of injuries in permanent teeth and 2-4% of injuries in primary teeth (4,5).

Root fracture is most important in permanent teeth. Root fracture healing is effect of location of fracture, type, root developing and distance of between fracture fragments. The fractures which occurred in the root due to trauma are classified as crown, medium and apical third based on their localization. According to fracture line, root fractures are classified as horizontal, oblique, vertical and horizontal/oblique (4).

Healing of root fractures was classified into four groups according to Andreasen and Hjørting-Hansen: (4).

1. Healing with hard tissue: contact points between fracture fragments are closed and fracture line disappeared or can only be traced very ambiguously.

2. Hard tissue and soft tissue accumulation between fracture fragments.

3. Healing with soft tissue accumulation between fracture fragments: radiolucent areas can be seen between fracture parts in radiography.

4. No healing: Because of pulp necrosis, radiolucent areas can be seen between fracture fragments and in alveolar bone in radiography.

In traditional and digital oral x-ray graphs, dental structures have seen in 2-D form. However, with Cone Beam Computed Tomography (CBCT) which recently is being used in dentistry, anatomic structure of the teeth and the mouth can be observed in any section and plane desired in a 3-D form. Thus, considerable advantage and convenience is obtained in diagnosis of occurred root fractures because of dental injuries (6-10).

Axial, coronal, sagittal and cross-sectional images can be obtained from the data provided by CBCT devices; as a result, it is now possible to identify in a more detailed and accurate manner fractures which are diagnosed or otherwise by traditional radiographies (9).

CBCT device provides to clinician with a wide area of usage for evaulation of dental fractures, cracks, measuring the size of periapical lesions, assessment of bone density in lesion area, endodontic surgery, implant planning, and analysis of temporomandibular joints and resorptions (11-14).

The dose of radiation is varies depending on the CBCT device, especially differences in scanning times and model and usage type of the device (15). The amount of radiation is directly related to the period of image taking and the field of image. For this reason, it is recommended that images are taken with as less field as possible and practicable. It imaging ensures clinicians with sub-millimeter spatial resolution images of high diagnostic standard with comparatively short scanning times (10–70 seconds) and a reported radiation dose same to that required for 4 - 15 panoramic radiographs (16).

The purpose of this study is to evaluate the CBCT image taken from 50 patients between 8-15 years of age who were subject to dental trauma.

## Material and Methods

In this study, CBCT images were taken and examined of 50 patients between 8 and 15 years of age were taken who applied to Dicle University, Faculty of Dentistry, Department of Pediatric Dentistry who were subject to trauma and suspected of dental root fracture. Before CBCT scans was taken, relatives of patients were informed about CBCT and the approval of the patient was obtained.

Only patients with anterior teeth fracture who were 8-15 years of age and healthy individuals were included in this study. The medical treatment history of patients who were included in this study was explored and information on the patients was collected by means of anamneses forms.

This study was confirmed by the ethical board of Dicle University, Faculty of Medicine on 8th December, 2011.

**Figure 1:**
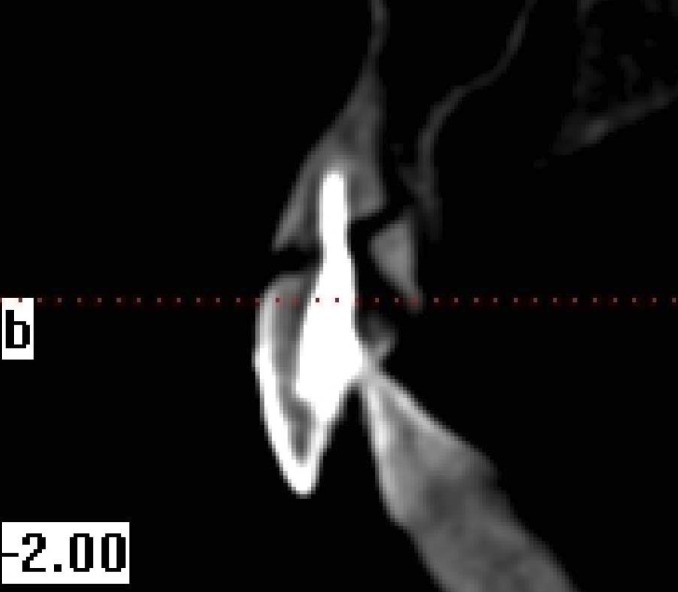
Cross sectional view oblique root fracture (right upper incisor tooth).

**Figure 2:**
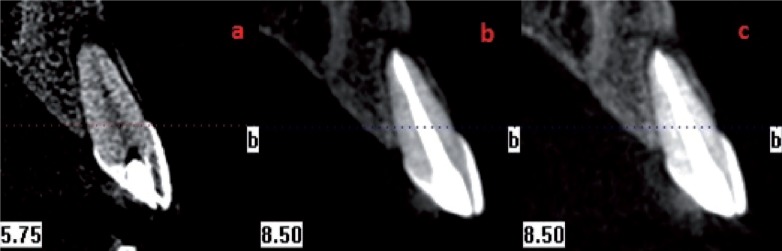
Apical 1/3 and horizontal root fracture. Pretreatment CBCT view (a), post treatment CBCT view (b), control CBCT view (c).

**Figure 3:**
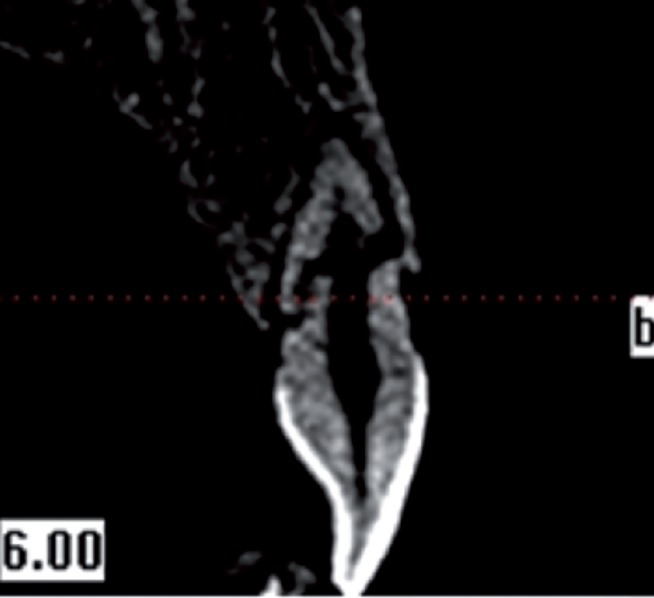
Fragmentary root fracture CBCT view.

**Figure 4:**
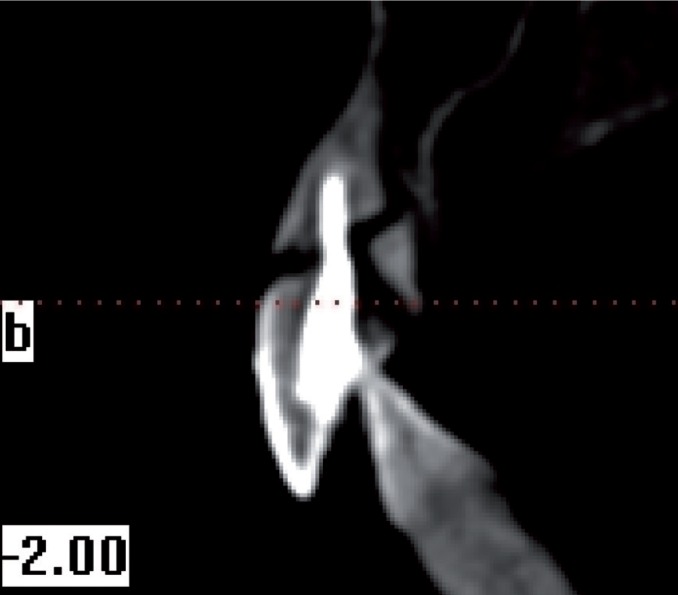
Apical 1/3 horizontal root fracture and cronal 1/3 vertical root fracture.

From all individuals included in this study, images were obtained in 8.9 seconds with CBCT device (i-CAT®, Model 17-19, Imaging Sciences International, Hatfield, Pa USA) (Figs. 1-4).

The teeth with post-trauma luxation were splinted. Generally orthodontic wire - composite splint was applied so that physiological movement of teeth could be allowed (0.5 mm full circle orthodontic wire, Kuraray 3M ESPE composite).

Endodontic treatments were applied to the teeth with pulp necrosis. In patients that include pulp but who did not experience pulp necrosis were treated with partial pulptomy and dental vitality was maintained.

In teeth with crown fracture, composite restoration was performed so as to reinstate aesthetic and function in relatively low damage cases (KURARAY 3M ESPE) and intra-canal post-aided restorations (fiber post, prefabricate post) were performed in relatively high damage cases.

## Results

Root fracture injuries were identified in a total of 97 teeth of the 50 children between 8 and 15 years of age who were exposed to trauma. It has been observed that frequency of root fractures was higher in boys (60%) compared to girls (40%). When the distribution of root fractures was examined based on age, it has been found out that injuries were more widespread in children 11-13 years of age. When the number of root fractures occurred in different age groups was examined, significant difference was observed between groups based on gender according to chi-square test ( Table 1, *P*<0.05 ).

**Table 1:**
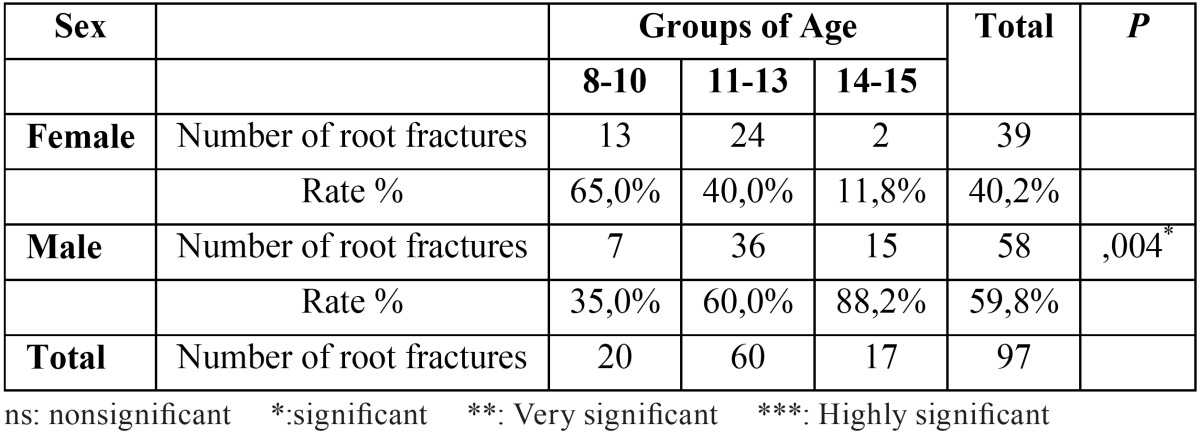
Investigation of age groups according to the number of root fracture.

 Table 2 gives the distribution and type of root fractures numbers obtained as a result of the evaluation of radiographic and CBCT images of the 50 patients.

**Table 2:**
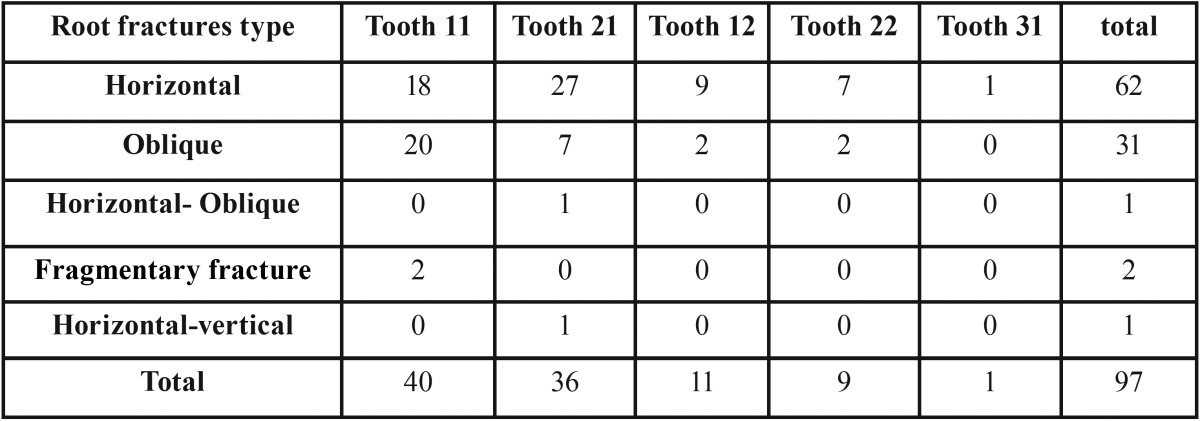
The distribution of 11, 21, 12, 22 and 31 of teeth occurred the root fractures.

As a result of more detailed analysis of the images obtained with CBCT, it has been observed that of the 97 teeth, 63.9% had horizontal root fracture, 31.9% had oblique root fracture, 1.03% had both horizontal and oblique root fracture, 2.06 had fragmentary fracture and 1.03% had both horizontal and vertical root fracture. An examination of the root fractures showed that 96 of the 97 teeth were maxillary teeth and 1 was mandibular tooth. The most affected teeth were determined as maxillary central incisor teeth (41.23% right 37.11% left), maxillary right lateral incisor teeth (11.34%), maxillary left lateral incisor teeth (9.27%) and mandibular central incisor teeth (1.03%) ( Table 2).

Fracture location of the 62 teeth which had horizontal root fracture, 11 teeth were at the middle region of the root, 4 teeth were in cervical region, and 47 were in the 1/3 apical region of root. Fracture of the 31 teeth which had oblique root fracture, 20 were in 1/3 apical region, 10 were in middle region and 1 was in cervical region. When the teeth with root fracture were examined as horizontal and oblique root fracture, according to chi-squaretest results, significant difference was found in the tooth no. 11 ( Table 3, 0.013* *P*<0.05).

**Table 3:**
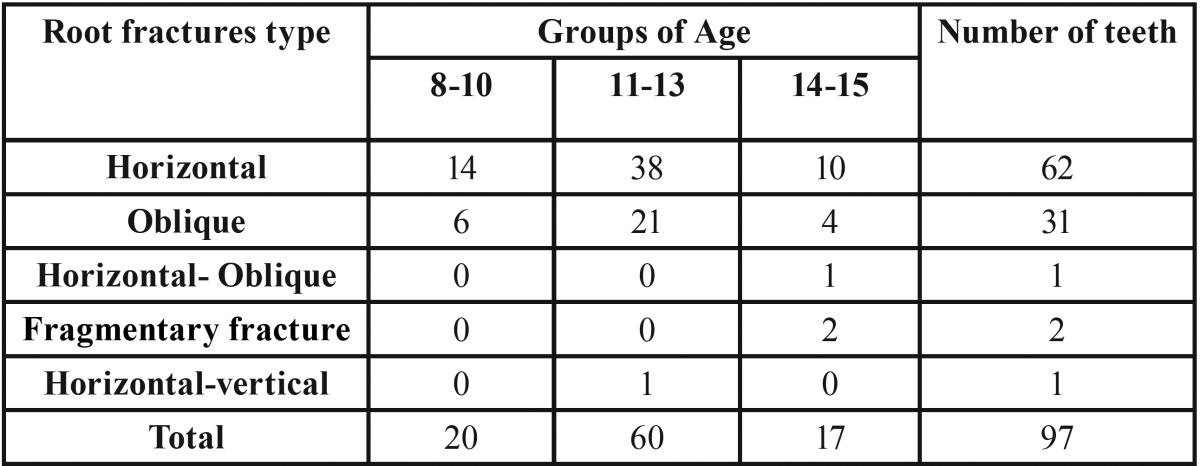
The distribution of fracture type and groups of age.

When the existence of crown fracture along with root fracture was taken into consideration, spontaneous healing rate was found to be higher in the absence of crown fracture. Statistically significant difference was also detected ( Table 4, *P*<0.05).

**Table 4:**
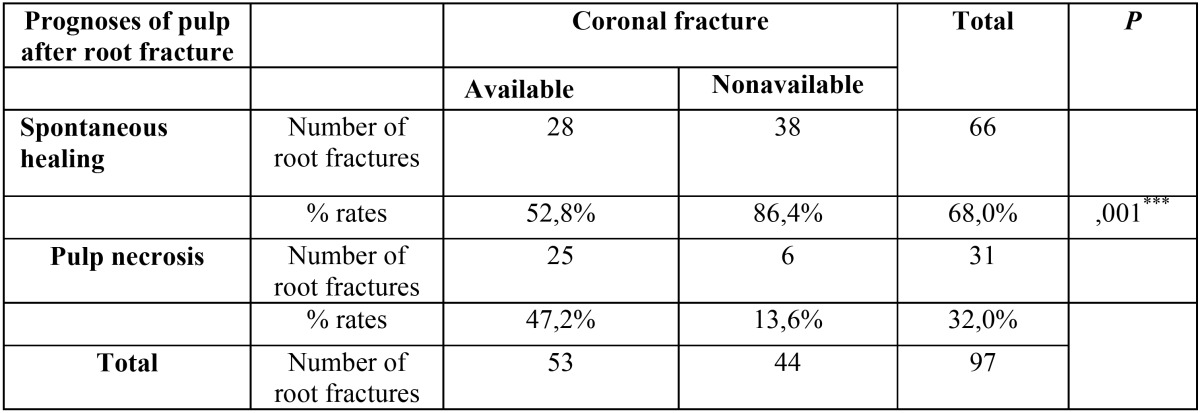
The distribution of pulp necrosis and spontaneous healing according to whether coronal fracture along with the root fracture.

## Discussion

Sudden and powerful impacts on teeth can cause root fractures, such as e.g. automobile accidents, sports injuries and fights. Dental injuries are most frequently observed in the maxillary anterior region (mainly central incisors) and reported to occur more frequently in maxillary teeth compared to mandibular teeth (9). Frequency of traumatic teeth injuries is mostly related to age and is reported to occur more frequently in the first 10 years of life. In the further years this frequency decreases and it is reported that dental injuries are less encountered at and above 30 years of age (17). Root fractures are most frequently observed in the 11–20 year age group male patients (18). In our study on 8-15 age intervals, it is determined that the highest rate of root fractures was in 11-13 age interval and in maxillary central incisors and male patients. Results of this study are consistent with the findings of other studies.

Root fractures are reported that occurrence mechanism of root fractures is usually in the form of a impingement from the frontal region and compression in labial and lingual or palatal direction (19). The injury in root fractures involves damage to pulp, dentin, cementum and periodontal ligament. Root fracture healing is effect of pulpal and periodontal healing processes and bacteria entering the coronal part of the pulp. Pulp necrosis was increased in enamel-dentin crown fracture teeth with extensive dentin exposure. Root fracture healing was significantly affected by pulp necrosis, dislocation fragments and luxation of the coronal fragment (4,19). In this study, the occurrence crown fracture along with root fracture had increased in incidence of pulp necrosis. In additionally, rate of spontaneous healing was increased in root fractures with the absence of crown fracture. It was significant difference found as per chi-square tests in the vital spontaneous healing and pulp necrosis ratios (*P*<0,05).

The some factors which had the important influence upon root fracture healing were: age, stage of root development, mobility and dislocation of the coronal fragment and distance of between the fracture fragments. The splinting procedures were usually absolute immobility in teeth with root fracture. Previous study reported that types of splinting were describes such as orthodontic band-arch wire splint, cap splint, composite splint, bonded metal wire splint and splinting with fiberglass. Rigid splinting could have a detrimental effect on root fracture healing (4). In this study, rigid splinting with composite was carried out only one patient, and it was developed pulp necrosis. For other patients, 0.5 mm fully circle orthodontic wire-composite was used for the purpose of splinting procedure. Splint was not carried out 49 teeth with root fractures which did not have luxation, had normal occlusion and mostly localised in apical 1/3. Spontaneous healing was observed on these teeth. These results are compatible with the results of a research conducted by Andreasen *et al.* (4).

Andreasen *et al.* reported that cervical fractures had observed pulp necrosis than fractures located at the middle or apical one-third of the root. In additionally, they said that optimal repositioning of root fractures with dislocation of the coronal fragment of up to 1 mm favors both healing with hard tissue and at the same time reduces the risk of pulp necrosis (24). In this study, apical 1/3 root fractures were observed spontaneous healing than fractures located at the middle and cervical. Furthermore, in one case, with more than 3mm dislocation of coronal fragment, was observed spontaneous healing after coronal fragment was repositioned and splinting procedure.

Horizontal root fractures occur the majority frequently in the middle of the root and infrequently in the apical 1/3 (18). In other study, apical and cervical 1/3 root fractures had equal frequency whereas middle of root fractures are more frequent (20,21). In our study, it was observed that root fractures were in apical 1/3 area in 68 teeth, middle of root in 23 teeth, in cervical 1/3 in 1 tooth and in more than one location in 5 teeth.

After fracture fragments are repositioned, splinting procedure is applied for a period of 4 weeks in middle of root fractures and 4 months in cervical 1/3 root fractures (18,20,21). The splinting period in our study was also applied in the same period.

Some researchers reported that apical 1/3 fractures cannot be detected in Clinique and periapical x-ray graphic, but cervical root fractures could be traced clearly (9,20). In our study, 3-D imaging was performed with the help of CBCT, thus, no problems were encountered in the identification of root fractures.

According to the epidemiological study performed by Çalışkan *et al.*, maxillary central incisor teeth were the most frequently affected 95 % of root fractures and most common location of root fracture was in middle 1/3 of the root (21,22). In our study, root fractures in maxillary central incisor, maxillary lateral and mandibular central teeth were found as 78.35%, 20.61% and 1.03%, respectively. These data obtained by us are compatible with the literature.

In radiographic evaluation of root fractures, it is recommended that periapical X-ray graph is taken with 45, 90 and 110 degrees angles (20). In another study it is recommended that occlusal graphs should also be taken along with x-ray graph (21). Another study stated that for identification of root fractures, the fracture line could be angled for which reason 2 or 3 x-ray graphs should be taken from different angles. It is also reported that identification of root fracture before a complication occurs which causes the loss of tooth such as post-traumatic root resorption, which could be best diagnosed by CBCT (23). In our research no problems were encountered in diagnosing root fractures as 3-dimensional imaging technique was being used.

The studies was reported that CBCT device was of great significance in diagnosis of dental root fractures and periapical lesions and, in addition to endodontic surgery, implant planning (11,14). It was also reported that mineralisation density which occurred with the healing in demineralised periapical lesion zones could be measured with HU ((Hounsfield units) scale (24). When all these researches are taken into consideration, it was believed that assessing root fractures with CBCT would be a good option and this study was conducted.

In conventional and digital oral radiographic three-dimensional anatomic dental structures can be viewed in 2-dimensional manner. On the other hand, CBCT devices can allow the clinician to identify in a shorter period of time root fractures which were diagnosed wrongly or not diagnosed at all with routine applications (9). In a study conducted on extracted molar teeth with CBCT and PR (periapical radiography), Bassam Hassan *et al.* found out that CBCT was superior in identifying vertical root fracture. They reported that in cases where x-rays did not pass from fracture lines, it would be difficult to identify root fractures with x-ray graph and examination of 3-dimensional dental structures would not be adequate. In the light of these informations, they stated that CBCT yielded clearer information than x-raygraph in identification of root fractures (6). Bernardes *et al.* compared conventional radiography and CBCT on 20 patients who received endodontic treatment with the suspect of root fracture and reported that CBCT was statistically superior to conventional radiography in identification of root fractures (7). Avsever *et al.* reported that in 2-dimensional radiographic images root fractures cannot be diagnosed accurately or cannot be diagnosed at all due to superposition of dental structures and anatomic formations (25). Micheal *et al.* compared CBCT and conventional radiography for assessing root fractures, as a result of which they reported that CBCT images were more superior to conventional radiography (26). In this study, teeth with real or suspected root fracture were examined in three-dimensional manner in three different planes and different cross-sections with CBCT. Detailed information has been obtained on location and type of fracture. Cases were examined from different cross-sections in axial, sagittal and coronal directions. In cross-sections from teeth of which image was taken, with images from different angles, more images and information was obtained compared to conventional x-ray graph. In cases which needed more information for treatment planning, CBCT has proved a very good guide and examination inspection tool. Particularly the cross-section images proved extremely beneficial in the identification of root fractures.

CBCT device can show differences in the amount of radiation that the patient is exposed to depending on the differences in scanning times, model of device and the length of irradiation used (25). At the same time, the amount of radiation is related to the thickness of voxel size. For this reason, it is recommended that as few cross-sections are taken as possible for diagnosis (26). In a study, it was reported that 0,3 voxel size was sufficient in dentistry practice and root fractures (27). In this study, CBCT was used to 0,3 voxel size and 8.9 seconds scanning time

Tomography allows for easier and more definite diagnosis in dental applications as it provides images at 3 planes and in 3 dimensions. In addition to the quality of obtained images and technical advantages of the device, another reason for preferring CBCT device over Multi-slice Computed Tomography-MSCT is that the patient is exposed to much lower radiation (26,28). Some researchers reported that the received radiation is 13 times lower (11,29,30). In addition, isotropic structure of the voxel ensures that the reshaped images are high quality and real-size, and it is reported that scanning was performed within 9 to 70 seconds (11,26). For these reasons, CBCT device is preferred for its lower radiation for patients compared to medical computed tomography.

Ludlow *et al.* stated in a study that CBCT scanning caused the release of 4 to 42 times more doses compared to panoramic examinations, but the dose given to the patient decreased as the size of the image area narrowed (31).

Current results show that CBCT device is a perfect option for identifying root fractures; however, there are some cases where usage of conic ray technology is limited. Today, CBCT device cannot be found in every dental clinique due to its high cost (32). Scanning performed with CBCT device decrease the artifacts in images compared to conventional computed topographies, but as for patients with radiopaque materials such as sealer, gutta-percha and metals which are light-proof, artifact formation can still be observed and the quality of scanning can be reduced, which is a remarkable point for the importance and necessity of taking symptoms in consideration in verification of diagnosis (33,34).

The effective dose from panoramic radiography is range from 5.5 to 22.0 μSv, cephalometric radiography 2.2-3.4 mSv, periapical radiography 1–8 mSv and occlusal radiography 8 mSv, and 11-77µSv in the area examined with CBCT device depending on the size, period and density of the area (35).

Although conic ray is an innovative and promising technology, effective radiation dose is higher compared to conventional oral and panoramic graphics. For this reason, it is considered that it is early to state that CBCT is a technique which should be used in all dental trauma cases. Today, CBCT must be considered as an alternative in cases when conventional radiography is insufficient in diagnosing root fractures.

## Conclusions

This study results showed that:

Root fractures occurred more often in male and maxillary central incisor teeth.

The rate of pulp necrosis was increased in teeth which occurred crown fracture along with root fracture.

As a result of obtained image with CBCT, according to location of root fractures, horizontal root fractures occurred more often than vertical and oblique root fractures.

Cross-sectional images gain advantage from diagnose and classification of root fractures.

Root fracture line could be in different shape when root fractures evaluated with CBCT.

In literature review, CBCT images were more superior to conventional radiography for diagnose of root fractures.
